# Multiphasic *On/Off* Pheromone Signalling in Moths as Neural Correlates of a Search Strategy

**DOI:** 10.1371/journal.pone.0061220

**Published:** 2013-04-17

**Authors:** Dominique Martinez, Antoine Chaffiol, Nicole Voges, Yuqiao Gu, Sylvia Anton, Jean-Pierre Rospars, Philippe Lucas

**Affiliations:** 1 UMR7503, Laboratoire Lorrain de Recherche en Informatique et ses Applications (LORIA), Centre National de la Recherche Scientifique (CNRS), Vandoeuvre-lès-Nancy, France; 2 UMR 1272, Physiologie de l'Insecte : Signalisation et Communication (PISC), Institut National de la Recherche Agronomique (INRA), Versailles, France; 3 Laboratoire Récepteurs et Canaux Ioniques Membranaires, UPRES-EA 2647 USC INRA 1330, Université d'Angers, Angers, France; Université Lyon, France

## Abstract

Insects and robots searching for odour sources in turbulent plumes face the same problem: the random nature of mixing causes fluctuations and intermittency in perception. Pheromone-tracking male moths appear to deal with discontinuous flows of information by surging upwind, upon sensing a pheromone patch, and casting crosswind, upon losing the plume. Using a combination of neurophysiological recordings, computational modelling and experiments with a cyborg, we propose a neuronal mechanism that promotes a behavioural switch between surge and casting. We show how multiphasic *On/Off* pheromone-sensitive neurons may guide action selection based on signalling presence or loss of the pheromone. A Hodgkin-Huxley-type neuron model with a small-conductance calcium-activated potassium (SK) channel reproduces physiological *On/Off* responses. Using this model as a command neuron and the antennae of tethered moths as pheromone sensors, we demonstrate the efficiency of multiphasic patterning in driving a robotic searcher toward the source. Taken together, our results suggest that multiphasic *On/Off* responses may mediate olfactory navigation and that SK channels may account for these responses.

## Introduction

The song “Should I stay or should I go?” [The Clash, 1982] deals with behavioural choices on the basis of internal states and sensory information, a fundamental property of all living creatures. For example, animals searching for mates switch between navigation strategies to cope with changing environments. In turbulent plumes that consist of intertwined odourized and non-odourized filaments [1], pheromone-seeking male moths alternate sequences of “go” (surge upwind while the odour is perceived), and “stay” (stop upwind progress if the odour is lost and zigzag crosswind until the plume is reacquired). This search strategy [2]–[6] is stimulus-driven in the sense that action selection is triggered by the current perception. The high velocity of flying insects imposes strong constraints on the reaction time [7], [8]. A rapid and reliable detection of presence and loss of the pheromone is crucial for the moth to engage an appropriate sequence of motor commands. Theoretical studies showed that decision-making is facilitated by using two types of neurons [9], one signalling the presence of the stimulus, the other one signalling its absence. Two distinct neurons responding either to an increase or a decrease in stimulus intensity have been found in cockroaches [10] with respect to food odours but not concerning pheromones. Currently, it is unclear how firing patterns in moths [11]–[12] encode presence and loss of the pheromone, *i.e.* stimulus *On* and *Off*, in order to guide action selection (“go” or “stay”).

In this work, we addressed this question by analyzing neural activities recorded in *Agrotis ipsilon* moths. We found that stimulus *On* and *Off* are not encoded in different neurons [9]–[10] but are, instead, time-multiplexed in the multiphasic response of single neurons. Typically, such multiphasic firing patterns are ascribed to synaptic interactions with GABAergic local neurons [11]–[12], but the experimental support for this hypothesis is rather ambiguous. Using a single neuron model, we here present another hypothesis, namely that such responses may arise primarily from an intrinsic calcium-dependent potassium conductance. Using this neuron model in robotic experiments, we then related these multiphasic responses to action selection.

## Results

### The Majority of MGC Neurons Exhibit Precise and Reliable *On/Off* Firing Patterns

**Figure 1:**
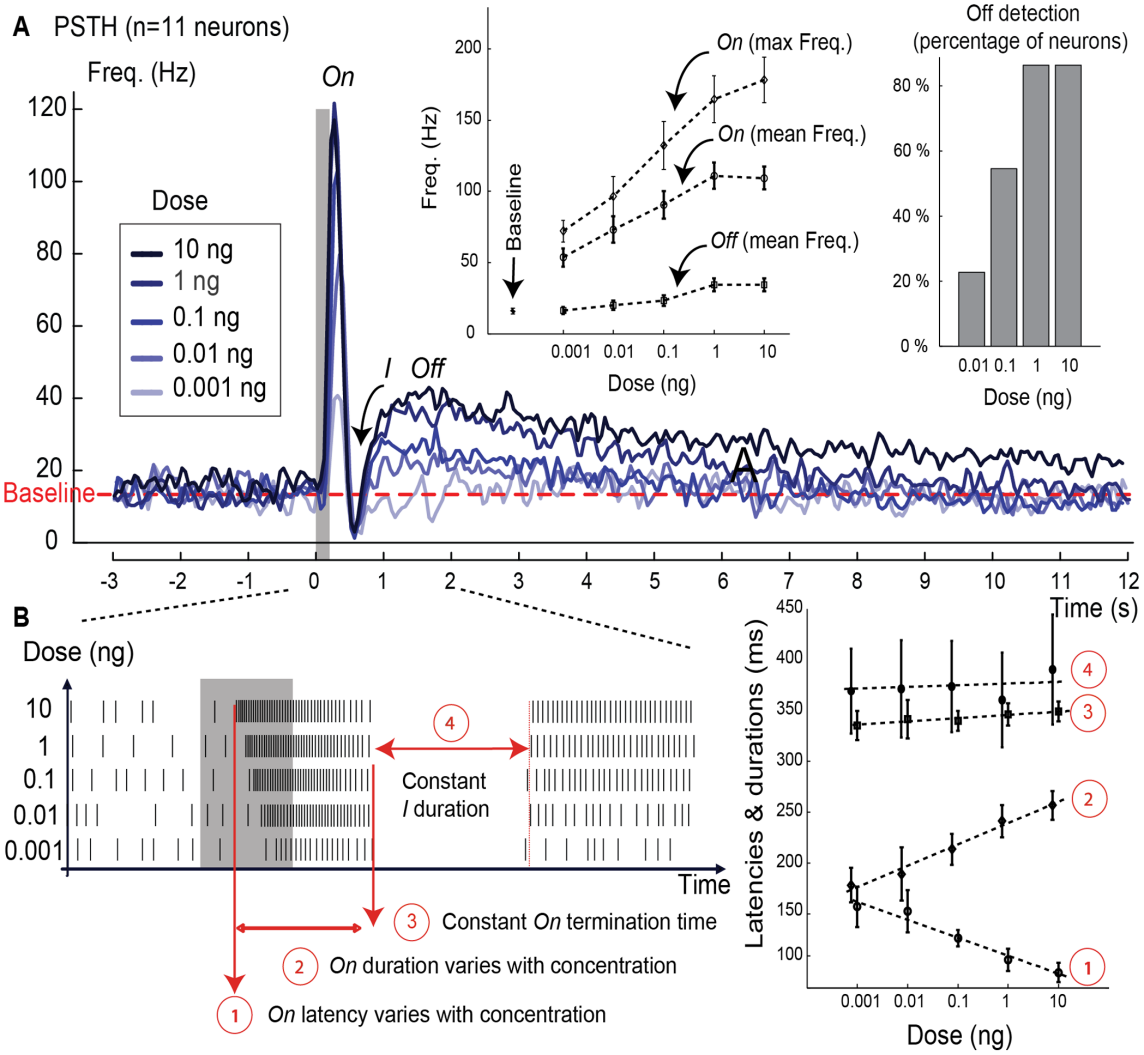
Dose effect on response frequencies and latencies. (**A**)**.** Peristimulus time histogram (PSTH) built using 11 neurons and a sliding window of 100 ms (50 ms overlap). The grey bar indicates the stimulus (pheromone blend of 200 ms at 5 different doses, from 0.001 to 10 ng). The dotted line represents the baseline activity (mean frequency). Figure inset at the left shows the *On* and *Off* firing frequencies at different doses (mean±s.e.m., *n = *11 neurons). The *Off* frequency was estimated by averaging over a one-second signal after the inhibitory phase. The *On* frequency (mean and max) was estimated using the complete *On* phase after stimulus onset. The value reported as baseline is the mean frequency of the spontaneous activity. At the lowest dose, *Off* and baseline have similar mean frequencies. Figure inset at the right shows that the percentage of the *Off* phase detected in the neural response increased with the dose (*n = *43 neurons). The presence of the *Off* was identified with the same segmentation method described for the *On* in Text S1. (**B**)**.** Effect of the dose on the latency and duration of the *On* and *I* phases (mean±s.e.m., *n = *11 neurons). The *On* latency decreased as the logarithm of the dose. The *On* duration increased as the logarithm of the dose. The *I* duration did not change with the dose.

We recorded neural activities extracellularly in the macroglomerular complex (MGC) of the moth *Agrotis ipsilon*, *i.e.* the specialist system processing pheromone information in the insect antennal lobe. The neurons responding to the pheromone (59 out of 74) were separated in two types based on their discharge patterns: monophasic (*n = *16, 27%) and multiphasic (*n* = 43, 73%). Multiphasic neurons were called *On/Off* neurons because their response to the pheromone was characterized by a prominent burst after stimulus onset (the *On* response, here called *On*), an inhibitory period (called *I*) and a long tonic excitation after stimulus offset (the *Off* response or *Off*), see Fig. 1A. Both *On* and *Off* responses depended on the stimulus dose (Fig. 1). The *On* latency, relative to stimulus onset, decreased according to -16 ms×log(dose/[ng]) +230 ms. The *On* duration increased correspondingly since its end was independent of the dose. The duration of the inhibitory phase did not change with the dose whereas the *Off* response was more often detected at higher doses.

**Figure 2:**
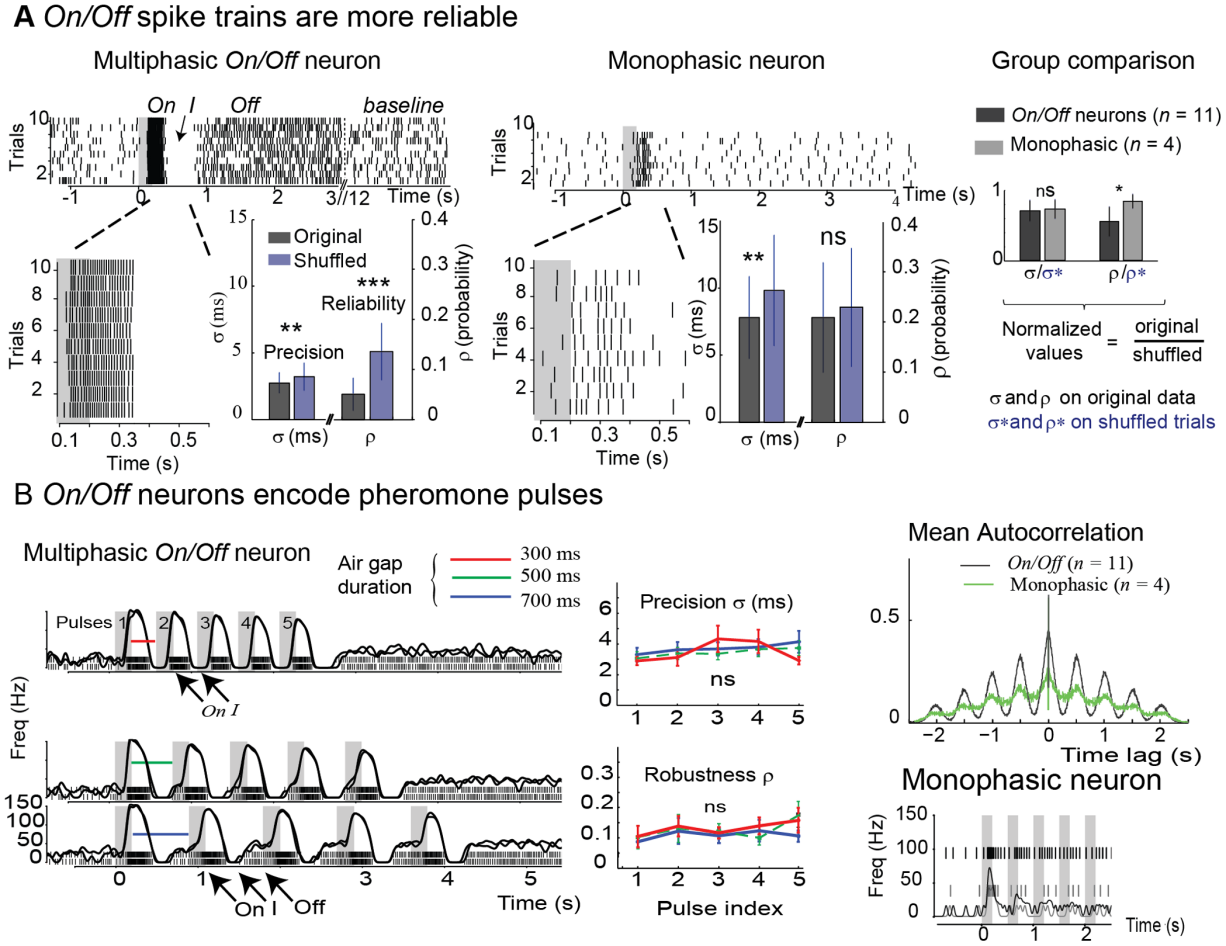
Precision and reliability of physiological responses. (**A**). Responses to single puffs of pheromone. Asterisks indicate significant differences between original and shuffled trials (*p<0.05, **p<0.01, ***p<0.001 and ns = no significant difference, Mann-Whitney test). Left: example of an *On/Off* neuron (73% of the recordings) over ten repeated trials (ticks depict individual spikes). Figure inset represents a zoom on the *On* response. The grey bar indicates the 200 ms stimulation period. The spike timing jitter σ (in ms) and the fraction of non-coincident spikes ρ were computed with the SES algorithm by considering all pairs of trials, *i.e.* 45 in total. Black bars represent σ and ρ obtained on the original spike trains and blue bars indicates σ* and ρ* obtained on shuffled trials (preserving interspike interval distribution). The *On/Off* neuron was both precise (σ<σ*) and reliable (ρ<ρ*). Middle: example of a monophasic neuron (27% of the recordings). The monophasic neuron was precise (p<0.01) but not reliable (p = 0.5, Mann-Whitney). Right: multiphasic versus monophasic neurons (*n = *number of neurons). To compensate for differences in firing rates, values were normalized as σ/σ* and ρ/ρ* for each neuron. (**B**) Responses to pulsed stimulations. Left: example of a multiphasic *On/Off* neuron exposed to 5 consecutive pheromone pulses of 200 ms separated by air gaps of 300, 500 or 700 ms. Each panel represents the spike trains from two repeated trials, superimposed with the Gaussian-convolved firing rate evolution. The *Off* phase is present after each pheromone pulses in the 700 and 500 ms air gap conditions and it is absent for higher frequency pulses (air gaps of 300 ms). Middle: precision and reliability across pulses in the different conditions. On average, σ = 3.6 ms and ρ = 0.1 (ns = no significant difference, Kruskal–Wallis test, *n = *11 neurons). Right: mean autocorrelation functions computed by averaging over monophasic and multiphasic *On/Off* neurons. Example of a monophasic neuron exposed to five consecutive pheromone pulses of 200 ms separated by air gaps of 300 ms.

We quantified *On* precision and reliability by the spike timing jitter σ (in ms) and the fraction of non-coincident spikes ρ computed over repeated trials. Neurons were considered to be precise for small σ and reliable for small ρ. Significance levels were determined by statistical comparisons with σ* and ρ* obtained on shuffled trials. In Fig. 2A, the *On/Off* neuron was both precise and reliable whereas the monophasic neuron was precise but not reliable. To compare precision and reliability between groups of monophasic (*n* = 8) and multiphasic (*n* = 11) neurons, we compensated for different firing rates, *i.e.* normalization according to σ/σ* and ρ/ρ* for each neuron (*Materials and Methods*). Again, ρ/ρ* differed between the two groups (Fig. 2A). Hence, *On/Off* neurons were more reliable than monophasic neurons.

### Pulsed Stimulations Preserve Precision and Reliability

*On/Off* neurons followed pheromone pulses up to several Hertz (Fig. 2B, 5 pulses of 200 ms pheromone separated by air gaps of 300, 500 and 700 ms). Note that the *Off* was absent between high frequency pulses (air gaps of 300 ms in Fig. 2B) and that the duration of the *Off* (respectively *On*) was well correlated with the duration of the air gaps (respectively pheromone pulses), (Fig. S1). The *On* was phase-locked to the stimulus (millisecond precision) and highly reliable, with σ and ρ not different, neither between successive pheromone pulses, nor between the different stimulation conditions (not significant, Kruskal-Wallis, *n* = 11). We computed the mean autocorrelation by averaging over monophasic (*n* = 4) and *On/Off* (*n* = 11) neurons. For *On/Off* neurons, we obtained periodic peaks separated by 500 ms corresponding to the period of the pulsed stimulus (Fig. 2B). For monophasic neurons, the autocorrelation was lower, thereby indicating a decline in tracking odour pulses (see also monophasic neuron in Fig. 2B).

### Bicuculline and Picrotoxin have Different Effects on Physiological *On/Off* Responses

**Figure 3:**
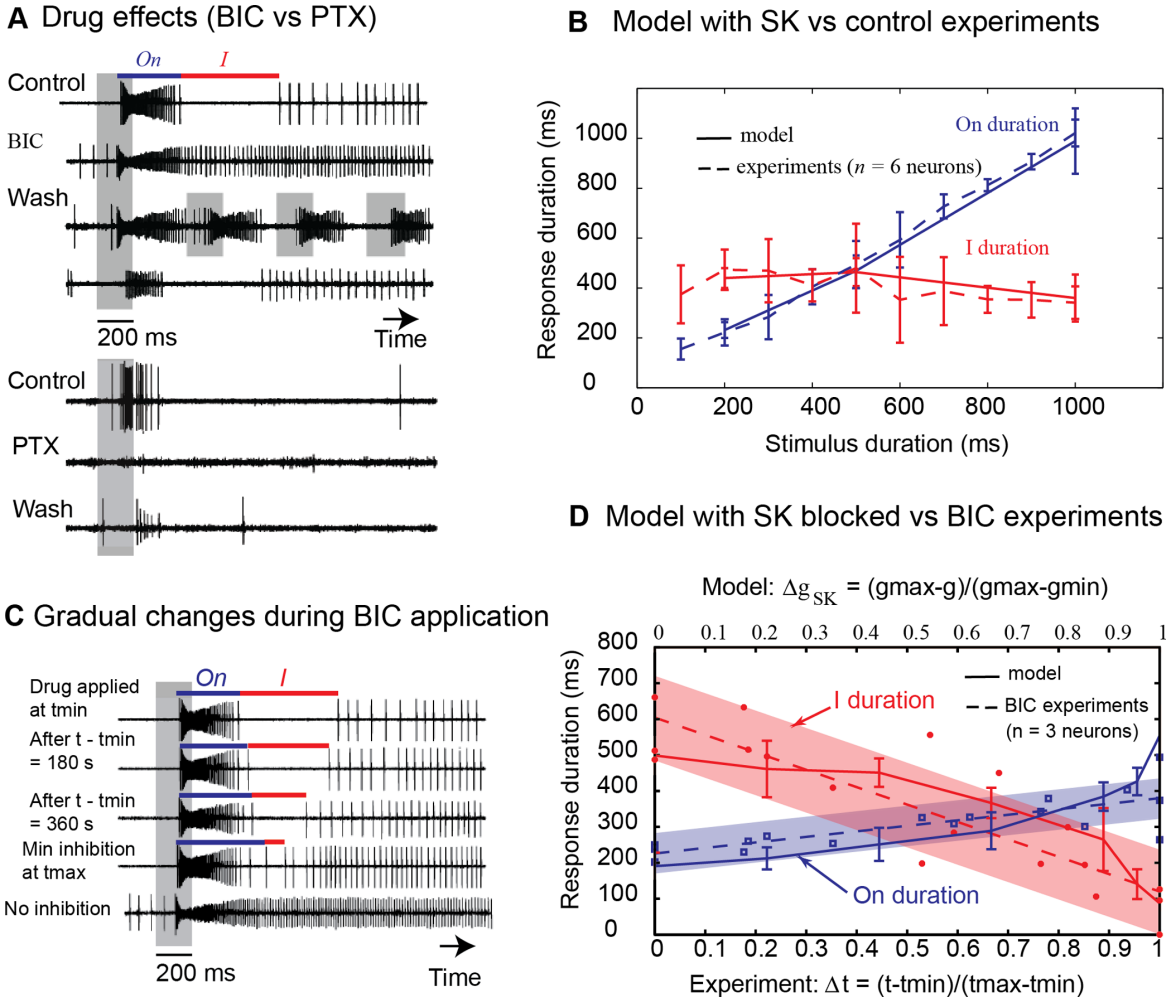
Pharmacological manipulations and neuron model. (**A**) Effects of bicuculline (BIC) and picrotoxin (PTX). Data are shown as raw traces. The stimulus (200 ms) is indicated by a grey bar. With BIC application (100 µM), the inhibitory phase was abolished so that the response to the pheromone changes from multiphasic to monophasic (*n* = 3 neurons). After wash-out, the multiphasic responses were recovered, as well as the ability to encode pheromone pulses. With PTX (100–250 µM), firing was suppressed during the spontaneous activity and the response to the pheromone (in 6 out of 7 neurons). The multiphasic responses were partially recovered after wash-out. (**B**) Simulation of the neuron model with SK versus control experimental data (dashed lines). *On/Off* neurons (*n = *6) were recorded for different stimulus durations (100 ms to 1 s, a single puff of the pheromone blend at 1 ng). The *On* duration depended linearly on stimulus duration: *On* duration = 0.99×(stimulus duration) +18 ms (Pearson correlation r^2^ = 0.97). The inhibitory phase was constant (p = 1, Kruskal–Wallis test): *I* duration = 399±106 ms. The neuron model (Text S1) was simulated with inputs mimicking a 1 ng pheromone blend stimulus (200, 500 or 1000 ms duration; 10 runs in each condition), resulting from fits of experimental data recorded in olfactory receptor neurons *in vivo* [12]. (**C**) Time dependent effect of BIC. Pheromone responses are shown at different times after BIC application (data as raw traces from the same neuron, stimulus (grey bar) = 200 ms). *tmin* indicates the start of BIC application (100 µM) and *tmax* indicates the time right before the inhibitory phase vanishes completely. (**D**) Simulation of the neuron model with SK blocked *versus* BIC experimental data. Data points represent *On* and *I* duration measured during BIC experiments (*n* = 3 neurons, stimulus duration = 200 ms), plotted *versus* the normalized time of BIC application (Δt at bottom axis, with *tmin* and *tmax* defined as in panel C). Dashed curves are linear fits of the data where blue and red envelopes show the 50% confidence bands. Plain curves represent *On* and *I* durations measured from simulations with partially blocked SK conductance. The durations of the two phases were plotted *versus* the normalized decrease in SK-like conductance (Δg_SK_ at top axis, with g ranging from *gmin* = 0.05 µS to *gmax* = 0.5 µS).

In pulsed stimulations, the inhibitory phase in *On/Off* neurons prevented long-lasting responses. The question arose as to whether it might be responsible for the ability to track intermittent stimuli. In *Manduca sexta*, the inhibitory phase was abolished by bicuculline (BIC), an antagonist of GABA_A_ receptors, which also disrupted the ability to encode pheromone pulses [13]. Here, we applied BIC (100 µM) and picrotoxin (PTX), another GABA_A_ antagonist, to *A. ipsilon* moths. BIC (100 µM) abolished the inhibitory phase in all tested neurons (*n* = 3, Fig. 3A). After wash-out, the multiphasic responses were recovered, as well as the ability to encode pheromone pulses. Unexpectedly, PTX (100–250 µM) led to the suppression of spontaneous activity and the complete loss of the response to the pheromone in 6 out of 7 neurons (Fig. 3A). Differences in BIC and PTX sensitivity can be explained by the fact that BIC, unlike PTX, has been shown in other species to block a Ca^2+^-dependent K^+^ channel, the small conductance (SK) channel [14].

### A Ca^2+^-activated K^+^ Conductance Neuron Model Reproduces *On/Off* firing Patterns

From the aforementioned pharmacological results, inhibition in *A. ipsilon* is presumably mediated by an SK conductance [15]. This hypothesis is difficult to test as the most common antagonist, apamin, is ineffective in insects [16]. Instead of digging further into pharmacological manipulation, we developed a biophysical neuron model (Text S1, Fig. S3). The objective was to investigate whether the experimentally observed *On/Off* responses can be reproduced with a Hodgkin-Huxley-type neuron model having an intrinsic SK conductance. With the SK conductance, inhibitory and *On* durations were in line with experimental data, *i.e.* inhibition ≈400 ms and *On* duration ≈ stimulus duration (Fig. 3B). Note that the correlation between *On* duration and stimulus duration emerged as a consequence of the model and was not explicitly taken into account during its development. In order to quantify how BIC affects the multiphasic response, we measured inhibitory and *On* durations in real neurons during bicuculline application (Fig. 3C). The time dependent effect of BIC was captured by linear fits (Fig. 3D, dashed lines) of the experimental data. It was in good agreement with the simulated model for partially blocked SK conductance (modelling results were within the 50% confidence band): the durations of the two phases were plotted versus the normalized decrease in SK conductance (Fig. 3D, plain curves). Thus, inhibition in *On/Off* neurons can well be mediated by an intrinsic Ca^2+^-dependent K^+^ conductance. Nevertheless, as cellular and network mechanisms are not mutually exclusive, these data do not rule out the implication of GABAergic local neurons.

### A Cyborg Driven by the Neuron Model Successfully Locates the Pheromone Source

**Figure 4:**
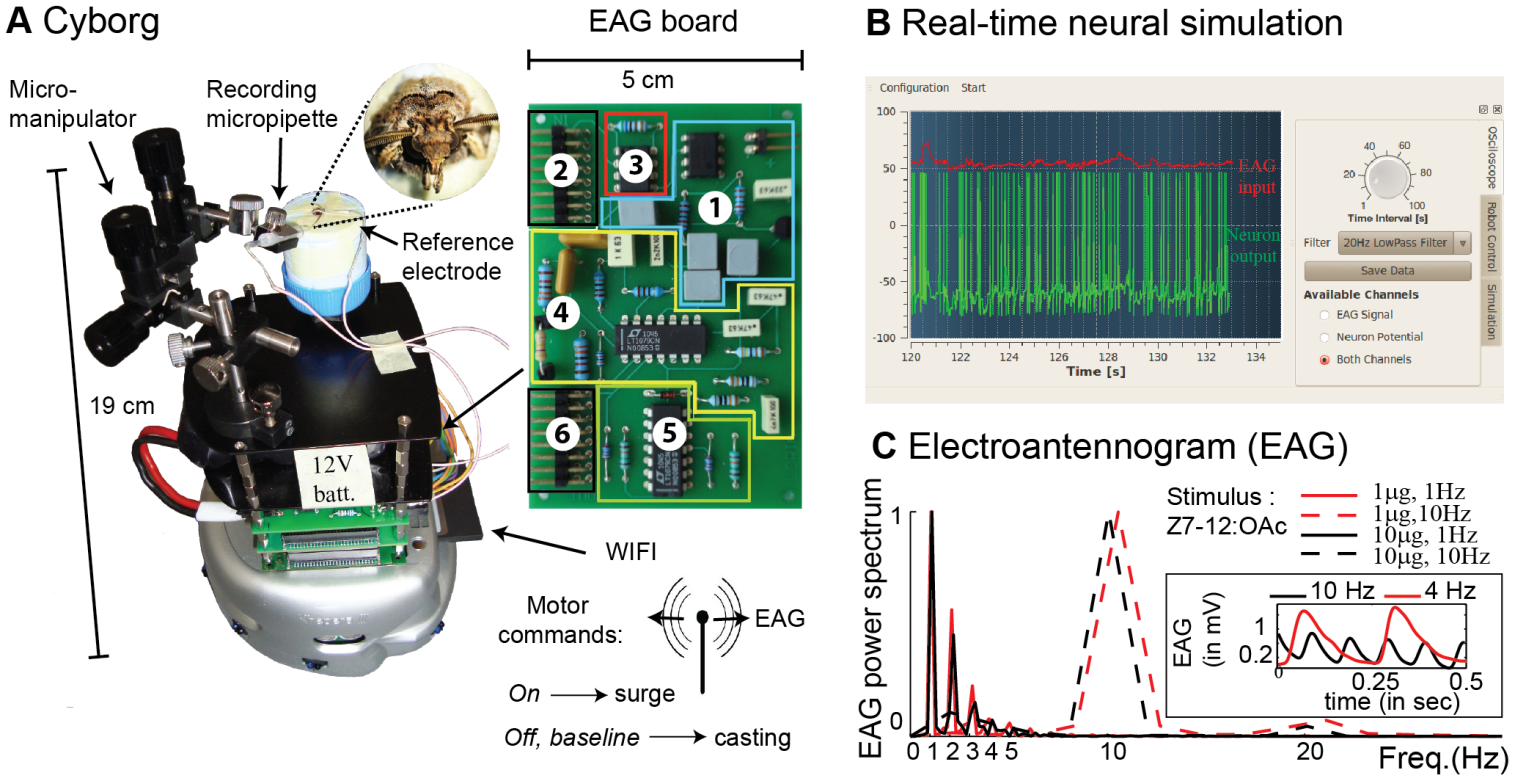
Cyborg experiments. (**A**) The robotic platform was composed of a tethered moth *A. ipsilon* mounted on a Khepera III robot. The EAG acquisition board consisted of (1) voltage regulation providing ±5 V from a +12 V battery, (2) differential EAG input, (3) instrumentation pre-amplification (INA121, ×10), (4) noise filtering and amplification (0.1–500 Hz frequency band, 50 Hz notch filter, ×25), (5) signal conditioning (0–5 V) and (6) analog-to-digital conversion (8 bits, 1 kHz sampling frequency). (**B**) Graphical user interface in Qt-C++ to visualize both EAG input and neuron output. The neuron simulation was performed in real-time. (**C**) The whole system was able to resolve pheromone pulses up to 10 Hz, as indicated by the normalized EAG power spectrum for pheromone puffs pulsed at 1 and 10 Hz. Inset: two examples of EAG signals obtained at 4 and 10 Hz.

**Figure 5:**
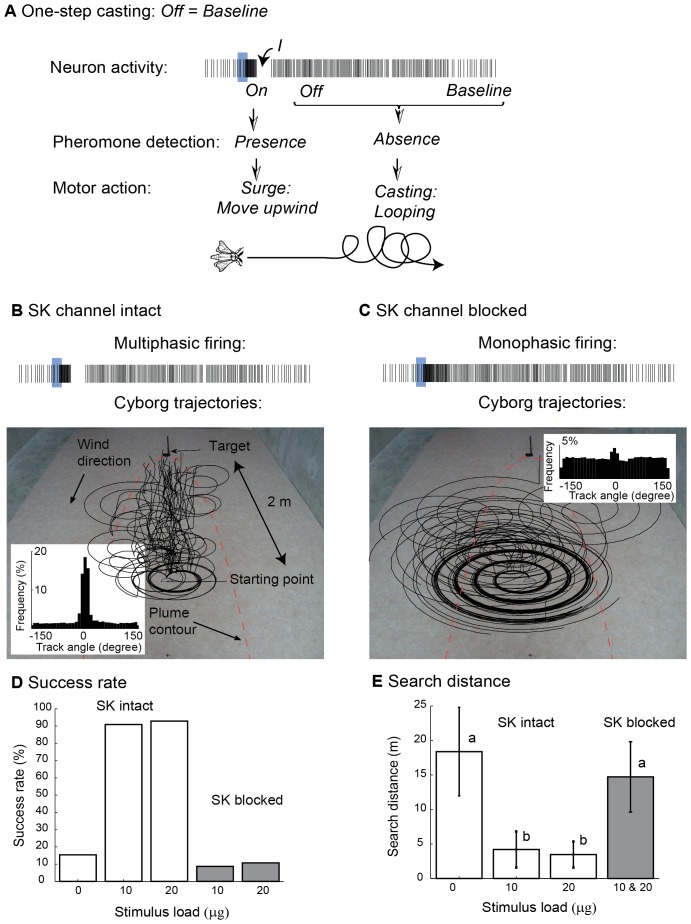
One-step casting. (**A**) In one-step casting, *Off* and baseline activities provide information about the absence of the stimulus and trigger the same casting behaviour: *Off*, *baseline →* spiraling. (**B**) Trajectories of the cyborg controlled by the *On/Off* neuron model with SK channel intact. The plume contour (red line) is defined as the parabolic region where 90% of all pheromone detections occurred. The target is the source of pheromone (dose = 10 µg). Inset: track angle histogram (p<0.001, Rayleigh circular test of non-uniformity). Track angles were computed as movement vectors with respect to the wind direction. A peak at 0° indicates a tendency to move upwind, as compared to movements perpendicular to the wind direction (±90°). (**C**) Trajectories with SK channel blocked. Inset: track angle histogram (p = 0.6, Rayleigh circular test of non-uniformity). Same experimental conditions as in panel B. (**D**) Success rate measured as the percentage of successful trials in the different conditions (SK channel intact and blocked, pheromone dose = 10 and 20 µg; the dose 0 µg stands for no pheromone). (**E)** Search distance measured from the initial location to the target for all successful trajectories. Conditions having no letters in common are significantly different at *p*<0.05 (Mann-Whitney pairwise comparisons).

Behavioural studies of male moths in pheromone plumes revealed distinct actions: surge and casting [2]–[6]. We suggest that *On* and *Off* responses could trigger surge and casting, respectively. Testing this hypothesis would ideally require to record from *On/Off* neurons in freely moving animals, a very challenging or even impossible task. Instead, we switched to robotic experiments using our *On/Off* neuron model as command neuron and the antennae of tethered moths as pheromone sensors (Fig. 4A). Antennae provided long lifetime and high sensitivity. With an initial distance to the source of 2 m (as compared to 10 cm in [17]) and a pheromone dose of 10 µg (as compared to 10 mg in [18]), pheromone detections became sporadic, similar to the situation in the field. The electroantennogram (EAG) system implemented on the robot resolved individual pheromone pulses up to 10 Hz (Fig. 4C). It was used as real-time input to our *On/Off* neuron model (a new input every millisecond, Fig. 4B). The surge command (straight movement in upwind direction) was triggered every time the *On* phase was followed by inhibition. The difficulty was to specify the casting. Initially, we considered a one-step casting strategy in which *Off* and baseline activities produced the same spiral behaviour (Fig. 5A). Without memory or clues about the search direction, spiralling is a secure search strategy, known to be used by other insects [19]. Search game theory [20] predicts that, if the searcher detects at plume centerline and nowhere else, then spiral casting combined with upwind surge results in a maximum search distance of 22.513×d, where d is the shortest initial distance to the source (Text S1, Fig. S4). A video illustrating the experiments (163 in total) is appended as Movie S1. With a SK channel in the neuron model, the success rate was 96% (Figs. 5B and 5D) and the search distance was 4±2 m (Fig. 5E). The track angle histogram (Fig. 5B, inset) had a peak at 0° (p<0.001, Rayleigh circular test of non-uniformity), indicating a predominance of the robot to move upwind. Track angle histograms with mode at 0° are also representative of moths flying upwind in turbulent plumes [13], [21]–[22]. Without SK in the neuron model, the robot located the source in 9% of the trials (Figs. 5C and 5E). The search distance was 15±5 m (Fig. 5E), indicating a predominance of the robot to perform spiral casting (Fig. 5C). The track angle was uniformly distributed (p = 0.6, Rayleigh test, Fig. 5C, inset). These results are well in line with the observations that BIC injection in moths severely diminished the track angle mode at 0° and prevented pheromone navigation [13].

### Combining Information Across Neurons Reduces the False Detection Rate

We noted that, without pheromone, the robot occasionally arrived at the source due to false detections (Fig. 5D). How reliable is then pheromone detection based on the activity of a single neuron? We addressed this question by performing a receiver operating characteristic (ROC) analysis on spike trains recorded in *A. ipsilon* moths (Text S1). At low pheromone doses, the detection was not robust, *e.g.* 90% of correct detections at 0.01 ng generated 15% of false alarms. We therefore recorded small populations of 2–3 neurons and repeated the ROC analysis. We observed synchronization on a millisecond time scale during the *On* phase which strengthened sensory decisions and reduced the likelihood of false detections (Text S1, Fig. S2). Similarly in robotic experiments without pheromone, the number of false alarms per trial reduced significantly when the robot was controlled with two-neuron rather than single-neuron activity: 1.14±1.23 (*n* = 44 trials) versus 3.21±2.75 (*n* = 14 trials, p<0.05, Mann-Whitney test).

### Two-step Casting is More Efficient

**Figure 6:**
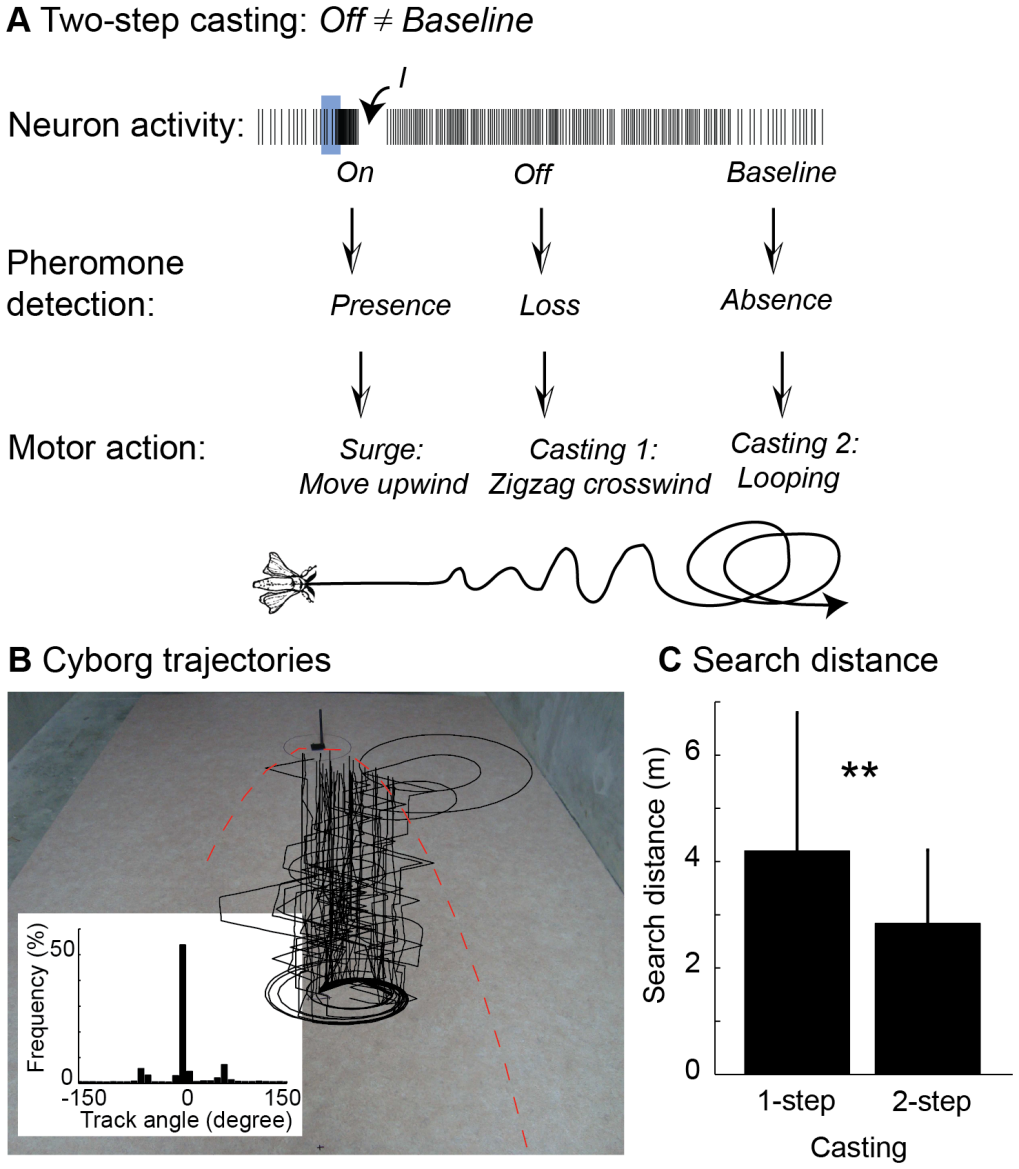
Two-step casting. (**A**) In two-step casting, *Off* and baseline activities trigger distinct casting behaviours: *Off →*crosswind zigzagging, *baseline →*spiraling. In this view, baseline activity provides information about the absence of the stimulus whereas the *Off* represents a recent sensory memory, indicating that the pheromone plume had been just lost. (**B**) Trajectories of the cyborg obtained with the two-step casting strategy. Inset: track angle histogram (p<0.001, Rayleigh circular test of non-uniformity). Same experimental conditions as in 1-step casting (Fig. 5B). To allow real-time processing, the *Off* detection was not performed explicitly: we simply considered that the *On* was followed by the *Off*. (**C**) Search distance of 2-step versus 1-step strategy (p<0.01, Mann-Whitney test).

We further considered a two-step casting strategy (Fig. 6A), *i.e.* distinct casting mobility patterns depending on whether the pheromone plume had been just lost or was absent for a long time. Baseline activity provided information about the absence of the stimulus and triggered spiral casting. In contrast, persistent firing during the *Off* may represent a recent sensory memory, indicating that the odour has been encountered just a short time ago. A good strategy to relocate the plume centreline is then to search in a line perpendicular to the wind. Using search game theory, we formulated crosswind casting as a linear search problem for which the optimal solution is to zigzag alternatively to the left and to the right, doubling the path length in every step [20]. Combined with upwind surge, this zigzagging strategy guarantees a maximum search distance of 9.055×d (Text S1). The cyborg trajectories are depicted in Fig. 6B. The track angle histogram had two modes, approximately at ±90°, characteristics of crosswind zigzagging. Bimodal track angle histograms were also observed with moths flying crosswind right after losing the plume [21]–[22]. As expected from search game theory, two-step casting outperformed one-step casting in cyborg experiments (Fig. 6C, Movie S2): search distance = 3±1 m (*n* = 66 trials) versus 4±2 m (*n* = 33).

## Discussion

Our results provide a possible mechanistic explanation for the behavioural model originally proposed by Baker twenty years ago: a phasic response ( = *On* in our study) generates an upwind surge and a separate, tonic response ( = *Off*) activates the casting [5]–[6]. The duration of the different phases was well consistent with behavioural observations: the *On* increased with stimulus duration (Fig. 3B), in line with the surge duration in several moth species [4]; the inhibitory period ≈400 ms corresponded well with the latency to switch from surge to casting [23]; the *Off* persisted for several seconds after removing the stimulus, a duration compatible with long-term casting; the *Off* was absent for pulses of high frequencies (Fig. 2B), in agreement with previous studies showing that high stimulation frequencies promote sustained upwind flights with nearly no casting [6].

Surge and casting in moths differ in their orientation to the wind. Surge is clearly upwind. Casting consists in zigzagging crosswind right after losing the plume [2]–[6], followed by looping or spiralling behaviours [24], [25]. Prior to initial contact with the odorant, casting has been shown to be preferably downwind [26] or non-oriented [27]. To account for differences in casting, we devised a two-step casting strategy based on the separation between *Off* and baseline activities (Fig. 6A) and demonstrated its efficiency using robotic experiments and search game theory [20]. However, the transition from *Off* to baseline in physiological recordings was continuous rather than discrete (Fig. 1A). Thus, it will be interesting to modulate the spiral search so as to shift gradually from crosswind to non-oriented casting. We note that the projection of a logarithmic spiral onto the crosswind direction results in a zigzag locomotion resembling to the one observed in the flight of moths. Yet, a proper characterization of casting in moths and further comparisons between theoretical and observed search patterns will require additional experiments.

Olberg [28] and Kanzaki et al. [29], [30] claimed that surge and casting are instructed in the moth protocerebrum, the target area of AL projection neurons (PNs), by two types of descending neurons (DNs) exhibiting short- and long-lasting excitatory responses. Recently, Kanzaki [31] suggested that the prolonged excitation in DNs arose from neuromodulatory action of serotonin. We here propose another possible origin, namely their PN inputs: our *On*/*Off* activity patterns closely resemble the responses of the DNs and of anatomically identified PNs [12]. Yet, it remains to be shown if time-multiplexed *On* and *Off* information can be sorted out in separate downstream neurons. This sensory-motor decoding may involve complex neural processing and other sensory modalities.

By enhancing the contrast between *On* and *Off*, the inhibitory phase may play an important role in transferring surge-casting information to motor neurons. In *M. sexta*, BIC abolished the inhibitory phase and disrupted pheromone navigation [13]. In *A. ipsilon*, different GABA_A_ antagonists had divergent effects on neuronal activity: BIC changed the response from multiphasic to monophasic while PTX completely suppressed firing. Firing suppression could result from the inactivation of Na^+^ channels following the sustained depolarization induced by the blockade of GABAergic synapses with PTX (disinhibiting effect). The different effect obtained with BIC could be explained by considering 1) that a subunit of the GABA_A_ receptor is PTX sensitive and BIC insensitive [32], and 2) that BIC acts as an antagonist of small-conductance Ca^2+^-dependent K^+^ (SK) channels [14].

SK channels have been reported in many neurons as being responsible for the prolonged after-hyperpolarization that follows bursts of action potentials [33]. We devised a Hodgkin-Huxley-type neuron model with an intrinsic SK conductance that reproduced the requisite properties of the inhibitory phase (Fig. 3B). Connecting our neuron model to a real moth antenna on a robot produced successful trajectories (Figs. 5B and 6B), similar to those observed for moths in wind tunnel experiments [13], [21], [22]. Without SK channel, the multiphasic neuron became monophasic and the pheromone source was generally not found (Fig. 5C), just as reported in the case of moths treated with BIC [13]. Other models may explain the data equally well, for example by considering interactions with inhibitory local neurons [15] or by using a cognitive navigation strategy [34]. However, these models will necessarily be of a larger complexity than the one presented here. In our work, we followed an Occam’s razor approach and it is worth noting that a unique neuron with an intrinsic SK channel may produce multiphasic responses mediating odour tracking behaviour.

Our work provides testable predictions and suggests new experimental approaches. Recently, a unique SK gene (dSK) has been identified in the *Drosophila* genome [35]. Given that dSK is expressed in fly’s olfactory PNs [35] and that olfactory navigation in flies and moths presents certain similarities, but also differences [36], it will be interesting to investigate the role of SK in olfactory processing using a genetically tractable animal model like *Drosophila.* In moths, Ca^2+^-dependent K^+^ currents have been found in the PNs, but their type was not identified [37]. Our work suggests that they could be of the SK type, a prediction that could be tested with a combination of molecular biology and electrophysiology. Finally, we note that the search algorithm was successfully implemented in hardware using insect antennae as sensors and a computational model as command neuron. Perhaps the most direct implication of this neural-engineering technology is its potential use in security applications, as insects are known to be sensitive to toxic gases, explosives or drugs [38], [39] and odour discrimination can be performed from their EAG responses [40].

## Materials and Methods

### Electrophysiology (Recording and Data Analysis)

Experiments were done on 5-day-old male moths *A. ipsilon* Hufnagel (Lepidoptera: Noctuidae). MGC neurons were recorded extracellularly by means of glass microelectrodes filled with Tucson ringer as described earlier [41]. Moths were stimulated with a pheromone blend of three main components: *cis*-7-dodecenyl acetate (Z7–12:OAc), *cis*-9- tetradecenyl acetate (Z9–14:OAc) and *cis*-11-hexadecenyl acetate (Z11–16:OAc), in the ratio of 4∶1:4. Stimulation of the antennae was controlled by a stimulation device (CS35, Syntech). The signal was amplified (IDAC 2000 amplifier, Syntech), band-pass filtered between 0.3 and 5 kHz and sampled at 16 kHz. The activity of nearby neurons was recorded using the Autospike software (v3.7, Syntech). Spike sorting was achieved with the R-package SpikeOMatic [42]. All other data analyses and statistical tests were done in Matlab (The MathWorks, Inc., Natick, MA, USA). Unless specified otherwise, data are presented as mean±s.d. The *On* response was identified using the segmentation method described in Text S1. The spike timing jitter σ (in ms) and the fraction of non-coincident spikes ρ were calculated with the stochastic event synchrony (SES) algorithm [43]. Significance levels were determined by statistical comparisons with σ* and ρ* obtained on shuffled trials (shuffling trials destroys within-trial temporal correlations while preserving interspike interval distribution). To compare precision and reliability between groups of neurons, we compensated for different firing rates by computing σ/σ* and ρ/ρ*, checking for significant differences in the deviations from the reference (shuffled trials) rather than in the absolute values themselves. In pharmacological experiments, 100–250 µM picrotoxin and 100 µM bicuculline methiodide (both from Sigma-Aldrich) were bath-applied to the moth preparation.

### Neuron Model

A model of the *On/Off* response was developed on the basis of Hodgkin–Huxley equations. A detailed description of the model is provided in Text S1. Briefly, the model incorporated five currents: leak, K^+^, Na^+^, Ca^2+^ and SK. Time constant and steady state functions for activation and inactivation variables were adapted from published data. To ensure similar inputs in simulation and in physiological recordings, the model was driven with an input current mimicking the temporal response profile of olfactory receptor neurons. In the cyborg experiments, the model was simulated in real-time with the EAG signal as input. The ordinary differential equations describing the model were numerically integrated with a fourth-order Runge-Kutta method (time step = 0.01 ms).

### Cyborg Experiments

Tethered moths *Agrotis ipsilon* were mounted on a Khepera III robot (K-Team) and the EAG was recorded from a whole-insect preparation (Fig. 4A). The insect was immobilized inside a styrofoam block allowing the head to protrude. For electrical contact, the last 2–3 segments of one antenna were cut-off. The recording electrode, a glass pipette filled with (in mM) 6.4 KCl, 340 glucose, 10 Hepes, 12 MgCl_2_, 1 CaCl_2_, 12 NaCl, pH 6.5, was maintained in contact using a micromanipulator (Narishige, UN-3C). A silver wire inserted into the neck served as the reference electrode. An EAG acquisition board was developed and embedded on the robot with appropriate processing. The EAG signal was transmitted via WIFI to a remote computer (dual Core laptop 1.6 GHz running Linux) and used as input to the neuron model. Neuron simulation, pheromone detection and robot control were performed in separate threads. The neuron simulation was performed in real-time with SIRENE, a C-based neural simulator developed by our team and available at http://sirene.gforge.inria.fr. A graphical user interface was written in Qt-C++ to visualize both EAG input and neuron output. A surge command was transmitted to the cyborg after each detection of the *On* phase: 3 consecutive interspike intervals <70 ms followed by inhibition (interspike interval ≥350 ms). In the two step-casting strategy, we simply assumed that the *On* was followed by the *Off*: the surge (duration = 5 sec) was followed by zigzag crosswind (duration = 19 sec). For the experiments with two neurons, the surge was triggered whenever the two neurons detected simultaneously. To account for heterogeneity in the two-neuron population, 20% Gaussian noise was added to some parameter values (SK conductance and calcium time constant). The search was performed in an arena of 4 m long by 2.5 m wide with the cyborg’s speed maintained constant at 5.6 cm/s. The robot was assumed to have reached its goal at 20 cm from the source. The cyborg trajectories were recorded using path integration. In order to obtain comparable results, all reported trials were performed with the robot initially located at (x, y) = (0, 0) and the pheromone source placed at (0, 2) expressed in meters. The source consisted of 10 µl and 20 µl of a 1 µg/µl solution of Z7–12:OAc (main pheromone component for *A. ipsilon*) alternatively deposited on a paper filter. To minimize variations in pheromone release throughout the experimental sessions, the filter paper was replaced every 2 trials. A laminar wind field was created by a fan placed at (0, 7). The wind velocity was relatively constant (0.9±0.2 m/s measured at source location with hot wire anemometer Testo 425). The wind direction was the same in all experiments and was a parameter given in advance to the robot.

## Supporting Information

Figure S1 **Effect of stimulus and air-gap durations.** **(A)** We stimulated *On/Off* neurons (*n = *5) with different stimulus durations (a unique puff, stimulus duration from 100 ms to 1 s). *On* duration showed a linear dependence on stimulus duration (data are presented as mean±s.d.): *On* duration = 0.99×(stimulus duration) +18 ms (pearson correlation r^2^ = 0.97). **(B).** We stimulated *On/Off* neurons (*n = *7) with randomized series of pulsed stimuli (air gap durations from 100 ms to 5 s, stimulus duration = 200 ms). *Off* duration showed a linear dependence on air gap duration (data are presented as mean±s.d.): *Off* duration = 0.88×(air gap duration) –273 ms (pearson correlation r^2^ = 0.97).
(TIF)

Figure S2 **Pheromone detection with multiple neurons.** **(A).** ROC analysis using three *On/Off* neurons recorded simultaneously (pheromone pulses of 200 ms, doses from 0.001 to 1 ng). Left: ROC curves calculated for single neurons as well as pairs and triplets (pheromone dose = 0.01 ng). Performance increases when the ROC curve is towards the left corner of the ROC space which corresponds to the ideal detector. Right: examples of spike trains used for the ROC curve calculations. The area under the ROC curve increased with the pheromone dose and the number of neurons. **(B).** Synchronized *On* activity. Five pairs of neurons were exposed to 5 consecutive pheromone pulses of 200 ms separated by air gaps of 300, 500 or 700 ms. Left: precision (σ = 3.43±1.38 ms, mean±s.d) across neurons in the different conditions (not significant, Kruskal–Wallis test). Right: robustness (ρ = 0.07±0.04, mean±s.d) across neurons in the different conditions (not significant, Kruskal–Wallis test).
(TIF)

Figure S3 **Simulation of the neuron model.** **(A).** ORN population model considered as a non-homogeneous Poisson process with rate parameter λ(t). The population firing rate λ(t) was derived from experimental data *(12)*. The instantaneous firing rate of 42 ORNs recorded for a stimulus dose of 1 ng and stimulus durations of 200 ms, 500 ms and 1 s was fitted as a sum of exponentials. Following stimulus onset at t = 0 s, λ(t) has three phases: rise, adaptation and decay. **(B).** The *On/Off* neuron model was simulated with the ORN population model as input for stimulus durations of 200 and 500 ms (stimulus onset indicated by the star).
(TIF)

Figure S4
**casting and search game theory.** Casting-surge is decomposed into a casting path 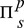 (in red) and a surge path 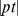 (straight line in black from *p* to *t*). **A.** If no direction information is available, spiral-surge achieves a competitive ratio *r* = 22.513. **B.** Given that the target is not downwind, zigzagging-surge achieves a competitive ratio *r = *9.0554.(TIF)

Text S1 **Supporting text S1 includes: Segmentation of the** ***On*** **phase in firing response patterns, Receiver operating characteristic (ROC) analysis,** ***On/Off*** **neuron model equations, Casting and search game theory, References.**
(PDF)

Movie S1 **One-step casting: SK intact vs SK blocked.** This movie shows two examples of the cyborg experiments with one-step casting: *On→*upwind surge, *Off* and *baseline→*spiral casting. The EAG input and *On/Off* neuron output are indicated in red and green, respectively. The movie contains two parts: channel SK intact and blocked.
(MOV)

Movie S2 **Two-step casting: SK intact.** This movie shows an example of the cyborg experiment with two-step casting: *On→*upwind surge, *Off→*casting 1 (crosswind zigzag), *baseline→*casting 2 (non-oriented spiral).
(MOV)
