# Supplementary figures and images for: Multiphasic On/Off Pheromone Signalling in Moths as Neural Correlates of a Search Strategy
Source: PLoS One. 2013 Apr 17;8(4):e61220. doi: 10.1371/journal.pone.0061220 (PMC3629186; doi:10.1371/journal.pone.0061220)

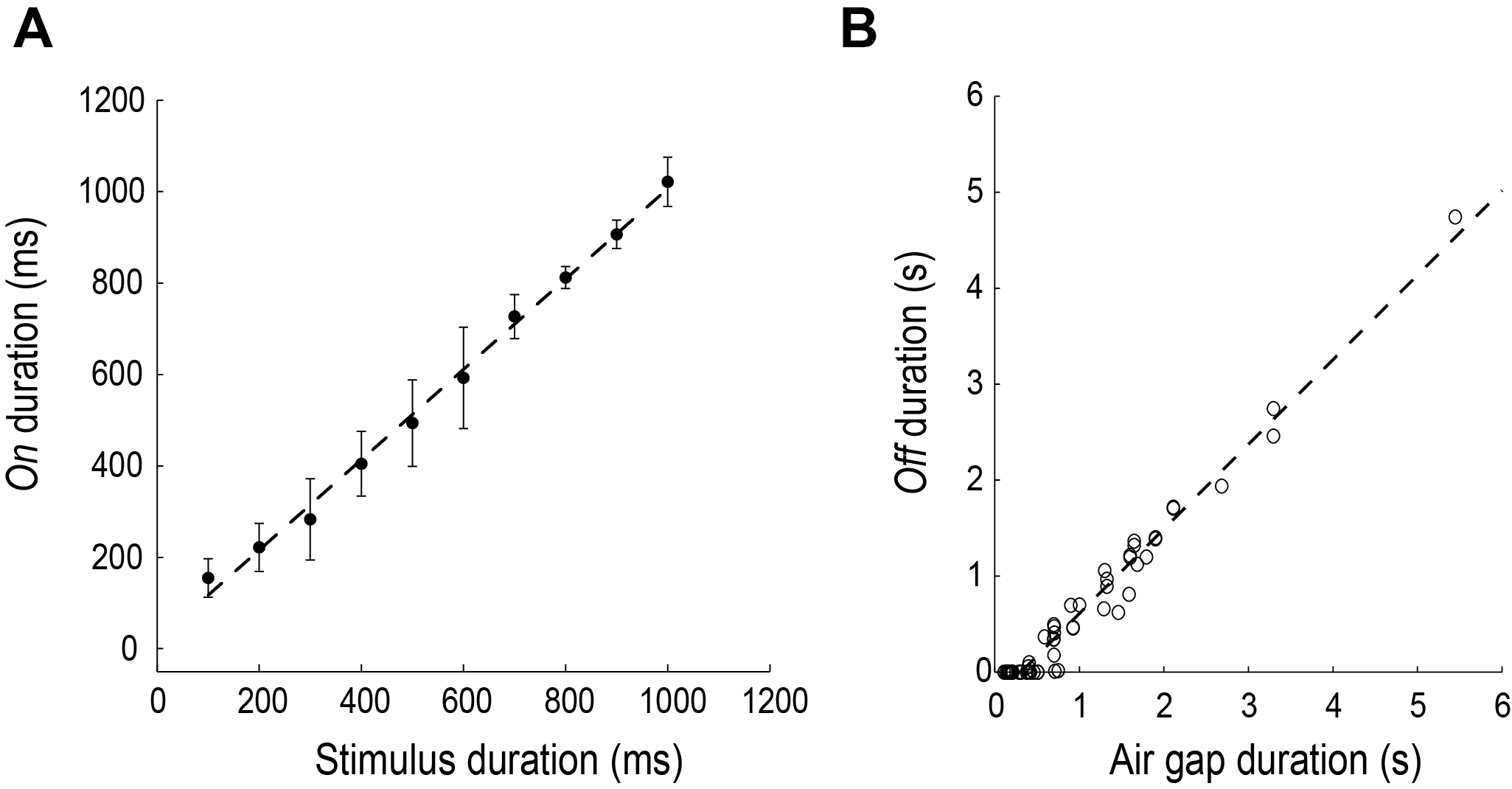

Supplement: Figure S1 — Effect of stimulus and air-gap durations. (A) We stimulated On/Off neurons (n = 5) with different stimulus durations (a unique puff, stimulus duration from 100 ms to 1 s). On duration showed a linear dependence on stimulus duration (data are presented as mean±s.d.): On duration = 0.99×(stimulus duration) +18 ms (pearson correlation r2 = 0.97). (B). We stimulated On/Off neurons (n = 7) with randomized series of pulsed stimuli (air gap durations from 100 ms to 5 s, stimulus duration = 200 ms). Off duration showed a linear dependence on air gap duration (data are presented as mean±s.d.): Off duration = 0.88×(air gap duration) –273 ms (pearson correlation r2 = 0.97). (TIF) [file pone.0061220.s001.tif]

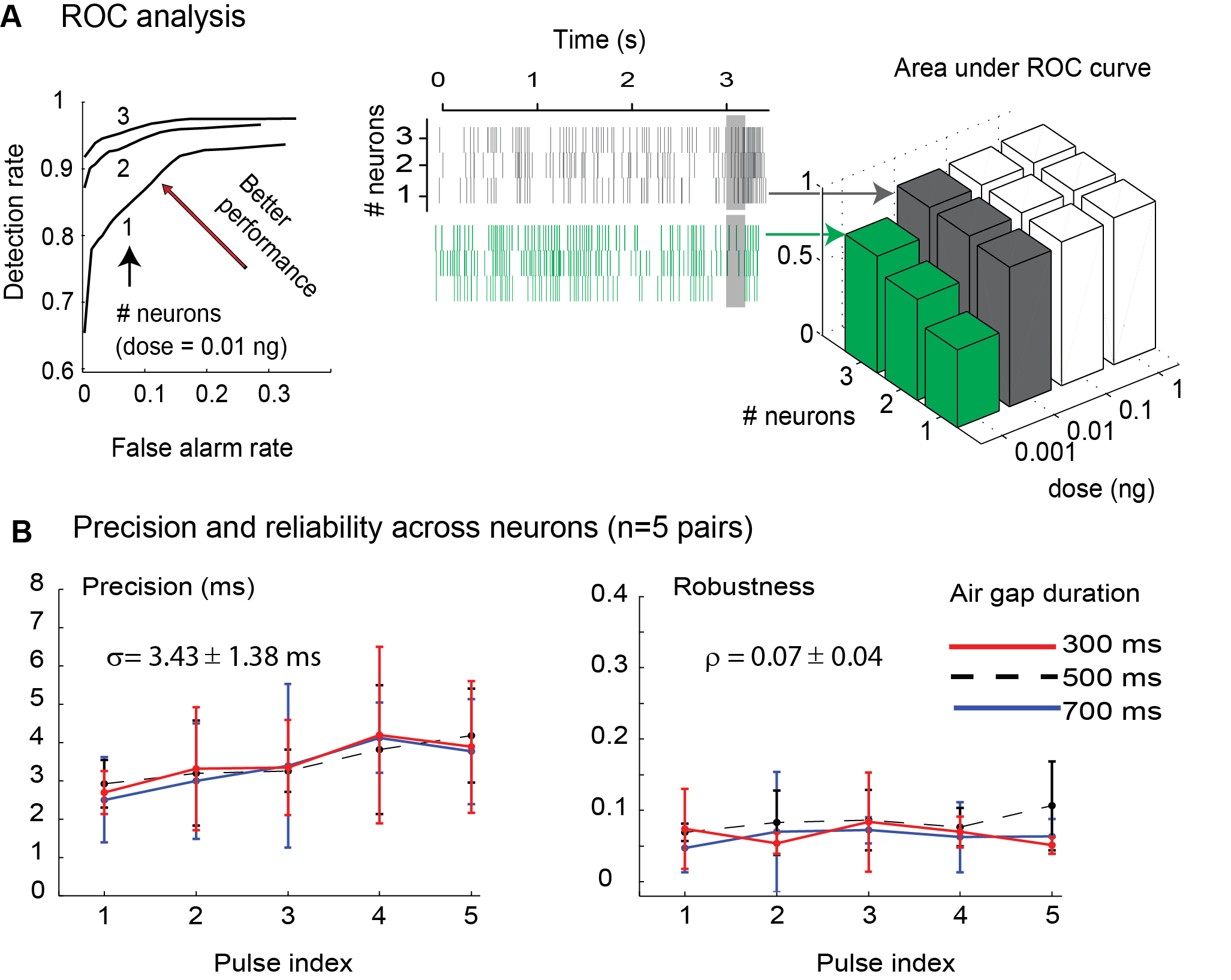

Supplement: Figure S2 — Pheromone detection with multiple neurons. (A). ROC analysis using three On/Off neurons recorded simultaneously (pheromone pulses of 200 ms, doses from 0.001 to 1 ng). Left: ROC curves calculated for single neurons as well as pairs and triplets (pheromone dose = 0.01 ng). Performance increases when the ROC curve is towards the left corner of the ROC space which corresponds to the ideal detector. Right: examples of spike trains used for the ROC curve calculations. The area under the ROC curve increased with the pheromone dose and the number of neurons. (B). Synchronized On activity. Five pairs of neurons were exposed to 5 consecutive pheromone pulses of 200 ms separated by air gaps of 300, 500 or 700 ms. Left: precision (σ = 3.43±1.38 ms, mean±s.d) across neurons in the different conditions (not significant, Kruskal–Wallis test). Right: robustness (ρ = 0.07±0.04, mean±s.d) across neurons in the different conditions (not significant, Kruskal–Wallis test). (TIF) [file pone.0061220.s002.tif]

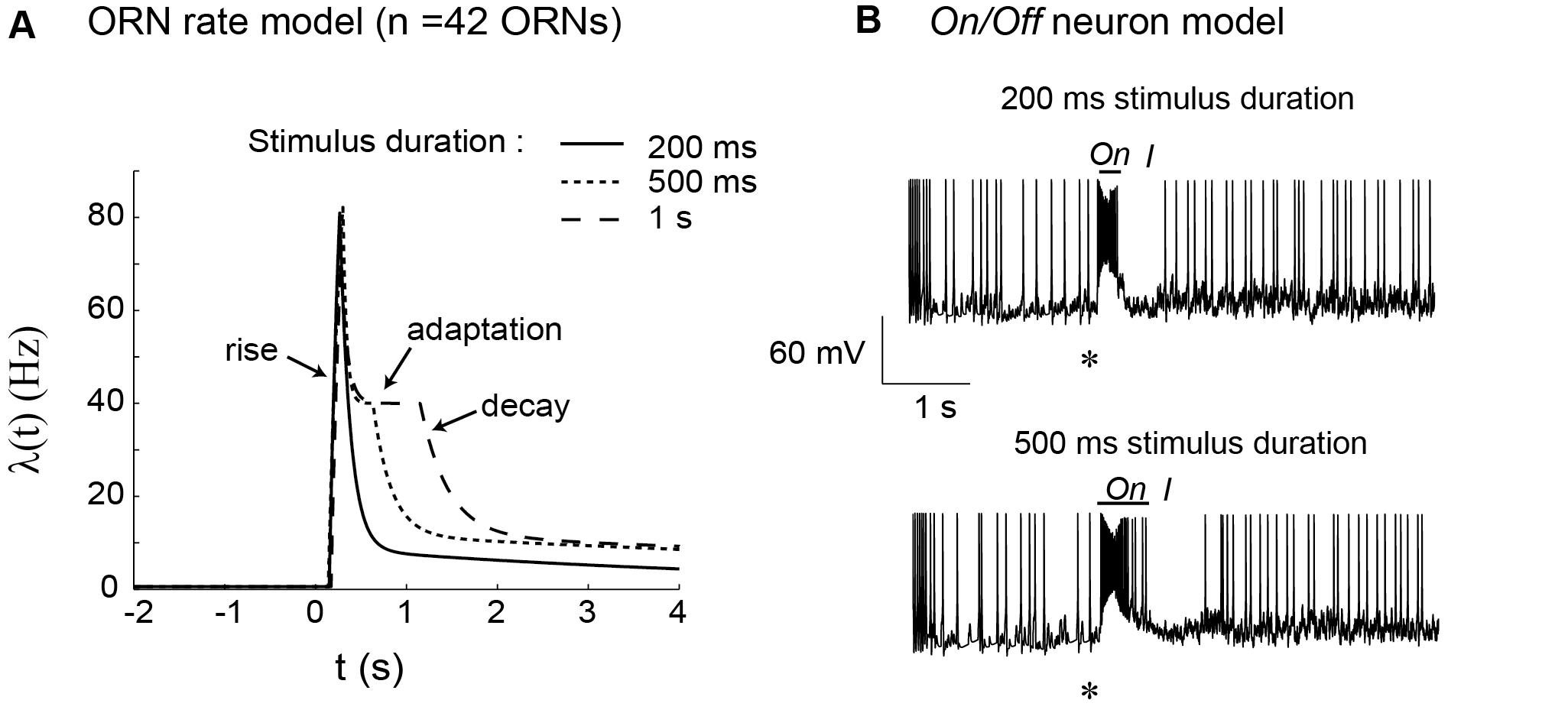

Supplement: Figure S3 — Simulation of the neuron model. (A). ORN population model considered as a non-homogeneous Poisson process with rate parameter λ(t). The population firing rate λ(t) was derived from experimental data (12). The instantaneous firing rate of 42 ORNs recorded for a stimulus dose of 1 ng and stimulus durations of 200 ms, 500 ms and 1 s was fitted as a sum of exponentials. Following stimulus onset at t = 0 s, λ(t) has three phases: rise, adaptation and decay. (B). The On/Off neuron model was simulated with the ORN population model as input for stimulus durations of 200 and 500 ms (stimulus onset indicated by the star). (TIF) [file pone.0061220.s003.tif]

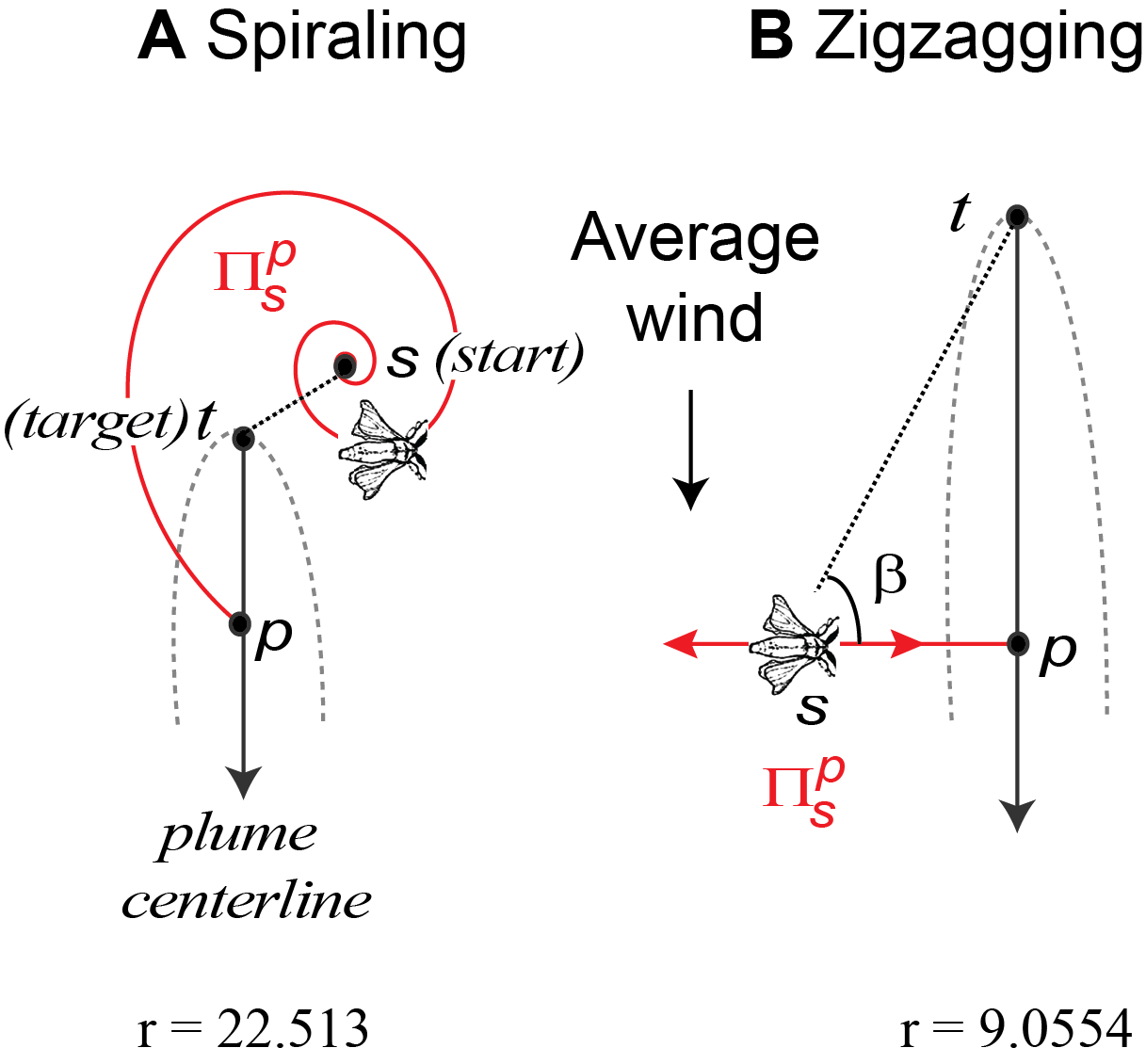

Supplement: Figure S4 — casting and search game theory. Casting-surge is decomposed into a casting path (in red) and a surge path (straight line in black from p to t). A. If no direction information is available, spiral-surge achieves a competitive ratio r = 22.513. B. Given that the target is not downwind, zigzagging-surge achieves a competitive ratio r = 9.0554. (TIF) [file pone.0061220.s004.tif]
