# Supplementary material for: Multiphasic On/Off Pheromone Signalling in Moths as Neural Correlates of a Search Strategy
Source: PLoS One. 2013 Apr 17;8(4):e61220. doi: 10.1371/journal.pone.0061220 (PMC3629186; doi:10.1371/journal.pone.0061220)
Supplement: Text S1 — Supporting text S1 includes: Segmentation of the On phase in firing response patterns, Receiver operating characteristic (ROC) analysis, On/Off neuron model equations, Casting and search game theory, References. (PDF) [file pone.0061220.s005.pdf]

**Text S1 includes:**

- Segmentation of the *On* phase in firing response patterns
- Receiver operating characteristic (ROC) analysis
- *On/Off* neuron model equations
- Casting and search game theory
- References

**Segmentation of the *On* phase in firing response patterns.** Following pheromone stimulation, the discharge pattern changed from spontaneous activity to the *On* phase. Interspike interval (ISI) distributions were well described by gamma densities (mean and coefficient of variation ± s.d.): $f_0$ ($\overline{ISI}$ = 85 ± 33 ms and *CV* = 0.8 ± 0.1) for the spontaneous activity versus $f_1$ ($\overline{ISI}$ = 10 ± 3 ms and *CV* = 0.34 ± 0.07) for the *On* ($n$ = 11 neurons, >6 trials per neuron). For every spike train, the change from $f_0$ to $f_1$ was detected using a CUSUM algorithm *(1-2)*. The log-likelihood of $f_1$ relative to $f_0$, based on a single observation (the $i$-th ISI), is

$$s(ISI_i) = \log\left(\frac{f_1(ISI_i)}{f_0(ISI_i)}\right)$$

The CUSUM algorithm allows processing consecutive ISIs by computing iteratively a cumulative sum of log-likelihood ratios

$$g_i = \max(g_{i-1} + s(ISI_i), 0) \qquad \text{(S1)}$$

with initial condition $g_0 = 0$. A change is detected as soon as $g_i \geq \theta$ (decision threshold = θ, *On* latency = time of detection). The end of the *On* was determined by the inhibitory period (ISI ≥ 100ms).

**Receiver operating characteristic (ROC) analysis.** According to signal detection theory, faster detection can be achieved at the expense of a higher false alarm rate and, vice versa, the more accurate the detection the higher the detection delay. Speed and accuracy can be traded by adjusting the decision threshold θ in the CUSUM detector (see above). A low θ will favour speed whereas a high θ results in a late but more accurate detection. We show that the speed-accuracy trade-off improves when considering the simultaneous activity of several neurons ($n$ = 3 *On/Off* neurons recorded simultaneously). The pheromone was detected as soon as the average cumulative sum $\frac{1}{N}\sum_{k=1}^{N} g_i^k$ for $N$ neurons exceeds the decision threshold θ ($g_i^k$ for the $k$-th neuron computed as in Eq. S1). The ROC curve *(3)* was estimated by computing detection and false

alarm rates for different thresholds (see in Fig. S2A the ROC curves obtained at 0.01 ng). Figure S2A shows that the area under the ROC curve increases with the dose and the number of neurons. The improvement in speed-accuracy trade-off resulting from using multiple neurons is probably due to correlated *On* activity. To assess the level of synchronization, we simultaneously recorded from 5 pairs of neurons (five pheromone pulses of 200ms, air gap of 300, 500 or 700ms). As shown in Fig. S2B, pulsed stimulations lead to rapid synchronization. The spikes emitted by different neurons were as precise and reliable as those produced by a single cell across repetitions ($\sigma$ =3.6 ms and $\rho$ =0.1, Fig. 2B in manuscript). Precision and reliability differed neither between successive pheromone pulses, nor between the different stimulation conditions (not significant, Kruskal-Wallis).

***On/Off* neuron model equations**. Our *On/Off* neuron model was a conductance-based model, with the membrane potential V governed by (Eq. S2)

$$C\frac{dV}{dt} = -g_L(V - E_L) - I_{Na} - I_{Kd} - I_{Ca} - I_{SK} + I_{ORN} \quad \text{(S2)}$$

The membrane capacitance C = 22.9 pF, leak conductance $g_L$ = 0.011161 µS and reversal potential $E_L$ = -61.4 mV were taken from *(4)*. Intrinsic sodium ($I_{Na}$) and delayed rectifier potassium ($I_{Kd}$) were described by (Eqs. S3-S5)

$$I = \overline{g}m^M h^N (V - E) \quad \text{(S3)}$$

$$\frac{dm}{dt} = (m_\infty - m)/\tau_m \quad \text{(S4)}$$

$$\frac{dh}{dt} = (h_\infty - h)/\tau_h \quad \text{(S5)}$$

where $m_\infty$ , $h_\infty$ , $\tau_m$ and $\tau_h$ are nonlinear functions of V. The $I_{Na}$ current was derived from data in *(5)*. It has M=3, N=1, $E_{Na}$ = 48.2 mV, $\overline{g}_{Na}$=9 µS, $m_{Na\infty} = 1/(1 + \exp((-15.8\text{-}V)/9.32))$, $h_{Na\infty} = 1/(1 + \exp((V{+}31.1)/9.75))$, $\tau_{mNa} = 0.19 + 2.17\exp(-(-23.33 - V)^2/(13.71)^2)$, $\tau_{hNa} = 1.57 + 8.83\exp(-(-29.15 - V)^2/(9.65)^2)$. The $I_{Kd}$ current was derived from data in (6-7). It has M=4, N=0, $E_K$ = -91.6 mV, $\overline{g}_{Kd}$=2.5 µS, $m_{Kd\infty} = 1/(1 + \exp((-18.5\text{-}V)/22.5))$ and $\tau_{mKd} = 1.62 + 6.93\exp(-(-33.65 - V)^2/(66.88)^2)$. The current $I_{Ca}$ was derived from data in *(4)*.
$I_{Ca} = \overline{g}_{Ca} m_{Ca} h_{Ca\infty}(V - E_{Ca})$ and $dm_{Ca}/dt = (m_{Ca\infty} - m_{Ca})/\tau_{mCa}$ with $E_{Ca}$ = 160 mV, $\overline{g}_{Ca}$=0.4 µS, $m_{Ca\infty} = 1/(1 + \exp((-10.6\text{-}V)/8.5))$, $h_{Ca\infty} = 1/(1 + \exp((V{+}29.6)/8.4))$ and $\tau_{mCa} = 1/(0.19\,((19.88 - V)/\exp(-1 + 0.1(19.88 - V)) + 0.046\exp(-V/20.73))$.
The current $I_{SK}$ was modified from *(8)*. $I_{SK} = \overline{g}_{SK} m_{SK\infty}(V - E_K)$ with SK conductance $\overline{g}_{SK}$=0.1 µS

and $m_{SK\infty} = 1/(1 + \exp(\text{-}1.12\text{-}2.508 \log((\text{Ca-}Ca_{\infty})/1000)))$. The calcium concentration evolved as $dCa/dt = 0.9\, I_{Ca} - (Ca - Ca_{\infty})/\tau_{Ca}$ with the calcium time constant $\tau_{Ca} = 900$ ms.

The current $I_{ORN}$ mimicked the total input received from a population of 100 ORNs. It was modelled as a sum of exponential decays $I_{ORN} = 0.02\text{pA} \times \sum_{i,f} e^{-(t-t_i^f)/\tau}$ where $t_i^f$ is firing time *f* of the *i*-th pre-synaptic ORN and $\tau$ =10 ms. The ORNs were considered to be non-homogeneous Poisson processes with the rate parameter λ(t) derived by fitting real ORN responses (Fig. S3A). The ordinary differential equations (S2-S5) were numerically integrated with a classical fourth-order Runge-Kutta method (time step = 10 µs). Simulations of the *On/Off* neuron model are shown in Fig. S3B.

**Casting and search game theory.** Tracking the pheromone source can be seen as a race with a single winner, that is, the fastest moth to reach the female. Casting should therefore be optimized so as to keep the search time, *i.e.* distance, as small as possible. We aimed at minimizing the maximum search distance (minimax strategy) and analyzed casting using search game theory *(9)*. Given that the searcher detects at plume centerline and nowhere else (deterministic search problem), the search can be decomposed into the following steps (Fig. S4): a casting path $\Pi_s^p$, starting from *s* and hitting the plume centerline in *p*, and a surge path $|pt|$, *i.e.* the straight line from *p* to target *t*. As *t* can be anywhere, the so-called competitive ratio *r* calculates the worst-case search distance in comparison to the shortest distance between *s* and *t* (Eq. S6).

$$r = \max_{t} \frac{\Pi_s^p + |pt|}{|st|} \qquad \text{(S6)}$$

If no direction information is available, *e.g.* before initial contact with the pheromone (Fig. S4A), the situation is similar to searching for the origin of a ray in the plane. There, spiral-surge achieves a competitive ratio of 22.51306… *(10)*. If prior information is provided by the average wind direction, *e.g.* after having lost contact with the pheromone (Fig. S4B), there is a more efficient strategy: relocate the plume centerline by casting in a line perpendicular to the wind. Crosswind casting can be formulated as a linear search problem for which the optimal solution is to zigzag alternatively to the left and to the right, doubling the path length in every step *(11)*. In the worst case, zigzag casting yields a search trajectory that is 9 times longer than the shortest distance between *s* and *p (11)*. The competitive ratio can thus be written as (Eqs. S7-S8)

$$r = \max_t \frac{9|sp| + |pt|}{|st|} \quad \text{(S7)}$$

$$= \max_\beta \cos\beta \left( 9 + \sqrt{\frac{1}{(\cos\beta)^2} - 1} \right) \quad \text{(S8)}$$

where the angle $\beta := \angle tsp$ depends on the position of *t* on the plume centerline (see Fig. S4B). The maximum is obtained for $\beta = 0.11065$ ... rads, leading to a competitive ratio *r* = 9.05538… Note that the minimum value of 9 is obtained for $\beta = 0$ rads, *i.e.* when *t* is in *p* so that the target is located with casting only.

**References.**


1. Basseville M. and Nikiforov I.V. (1993) Detection of Abrupt Changes - Theory and Application. Prentice-Hall, Inc. (Englewood Cliffs, N.J.)
2. Ratnam R., Goense J.B.M. and Nelson M.E. (2003) Change-point detection in neuronal spike train activity. Neurocomputing. 52-54:849-855.
3. Fawcett T. (2006). An introduction to ROC analysis. Pattern Recognition Letters. 27:861-874.
4. Husch A, Paehler M, Fusca D, Paeger L, and Kloppenburg P (2009) Calcium current diversity in physiologically different local interneuron types of the antennal lobe. J Neurosci 29(3):716-726.
5. Lapied B, Malecot CO, Pelhate M (1990) Patch-clamp study of the properties of the sodium current in cockroach single isolated adult aminergic neurons. J Exp Biol. 151:387–404.
6. Kloppenburg P, Ferns D and Mercer AR (1999) Serotonin enhances central olfactory neuron responses to female sex pheromone in the male sphinx moth *Manduca sexta*. J Neurosci 19(19):8172–8181.
7. Mercer AR, Hayashi JH and Hildebrand JG (1995) Modulatory effects of 5-hydroxytryptamine on voltage-activated currents in cultured antennal lobe neurones of the sphinx moth *Manduca sexta*. J Exp Biol 198, 613–627.
8. Roper P, Callaway J, T Shevchenko, R Teruyama and W Armstrong (2003) AHP's, HAP's and DAP's: How potassium currents regulate the excitability of rat supraoptic neurones. J. Comp. Neurosci. 15:367-389.
9. Alperin, S. & Gal, S. *The theory of search games and rendezvous* (Kluwer Academic Publishers 2003).

10. Eubeler, A., Fleischer, R., Kamphans, T., Klein, R., Langetepe, E. & Trippen, G. Competitive online searching for a ray in the plane. Dagstuhl Seminar Proceedings (Fekete, S., Fleischer, R., Klein, R. & Lopez-Ortiz, A. eds,, 2006).
11. Baeza-Yates, R., Culberson, J. & Rawlins, G. Searching in the plane. *Inform. Comput.* **106**, 234-252 (1993).
12. Jarriault D., Gadenne C., Rospars J.-P., Anton S. (2009). Quantitative analysis of sex-pheromone coding in the antennal lobe of the moth *Agrotis ipsilon*: a tool to study network plasticity. J. exp. Biol. 212:1191-1201.
